# Scrofuloderma and Bilateral Anterior Staphyloma of Eye: An Unusual Association

**DOI:** 10.4103/0974-9233.75894

**Published:** 2011

**Authors:** Asim K. Kandar, Rajesh Sinha, Namrata Sharma, K. Mathew James, Shveta J. Bali, Jeewan S. Titiyal

**Affiliations:** Rajendra Prasad Centre for Ophthalmic Sciences, All India Institute of Medical Sciences, New Delhi, India

**Keywords:** Anterior Staphyloma, Corneal Ulcer, Scrofuloderma

## Abstract

We report a patient with bilaterally symmetrical perforated corneal ulcers with formation of anterior staphyloma associated with scrofuloderma. A 22-year-old female in her third month postpartum presented with multiple perforated corneal ulcers bilaterally. There was a presence of a skin lesion that was consistent with scrofuloderma. Fine needle aspiration cytology of preauricular lymph nodes showed evidence of granulomatous inflammation consistent with tuberculosis. Corneal scraping revealed the presence of coagulase-negative Staphylococcus. She was treated with topical fortified combination antibiotics (cefazolin and tobramycin). The corneal ulcer resolved with formation of anterior staphyloma in both eyes. The patient underwent anterior *staphylectomy* and tectonic keratoplasty in both eyes. Eight weeks postoperatively, her visual acuity improved to 20/200 in both eyes with clear grafts. Scrofuloderma may be associated with recurrent phlyctenulosis. Multiple corneal ulcerations coupled with use of topical steroids may result in corneal perforation and formation of anterior staphyloma.

## INTRODUCTION

Tuberculosis is one of the oldest diseases known to mankind and continues to be a significant health problem even in the current century. Extrapulmonary tuberculosis constitutes 10% of the total tuberculosis burden. Of these cases, 1.5% have cutaneous disease.^1^ Ocular tuberculosis may occur in the absence of pulmonary disease. Early diagnosis and prompt treatment of this clinical entity may prevent ocular morbidity and blindness.^2^ Ocular involvement is well described in association with cutaneous tuberculosis; most reports documented are phlyctenular keratoconjunctivitis, tarsitis, and orbital tuberculosis.^3^ Ocular scrofuloderma with orbital involvement has been previously described.^4^,^5^ However, to the best of our knowledge, no case of bilateral simultaneous symmetrical multiple corneal ulceration with perforation has been reported in the English peer review literature.

In this report, we present a case of scrofuloderma associated with bilateral, multiple perforated corneal ulcers with formation of anterior staphyloma which required penetrating keratoplasty.

## CASE REPORT

A 22-year-old female presented with a history of redness, discharge, photophobia, blepharospasm, and progressive loss of vision over month in both the eyes.

She was 3 month postpartum with a self-reported history of three episodes of pain, redness and watering in both eyes in the antenatal period. The redness was localized to a different quadrant on every occasion. She was treated with a topical combination drop of gatifloxacin sesquihydrate (0.3%) and prednisolone acetate (1%) QID by her ophthalmologist. The redness and pain resolved within a week following each episode of initiating therapy.

She did not report any episodes of eczema, arthritis, chronic cough, recurrent fever, or exanthematous lesions. There was no history of ocular mechanical or chemical trauma.

She was living with her mother who was being treated for pulmonary tuberculosis.

**Figure 1:**
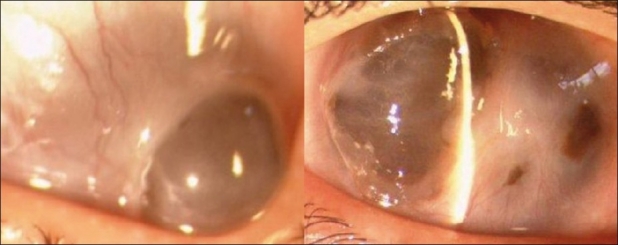
Right and left eye respectively showing multiple corneal perforations at presentation eventually resulting in the formation of anterior staphyloma

The visual acuity in both eyes was light perception with inaccurate projection. Mucopurulent discharge with circumcorneal congestion was noted in both eyes. Slit lamp examination revealed corneal infiltration with four areas of corneal perforation in the left eye (measuring 5.2 mm ×5.5 mm, 3.3 × 2.7 mm, 2.2 mm × 2.6 mm, 1.8 mm × 1.6 mm) and two areas of perforation (measuring 5.8 × 6.2 mm and 2.2 × 2.4 mm) in the right eye with pseudocornea formation and a flat anterior chamber. The lesions were peripheral encroaching on the paracentral and central areas [Figure 1]. Lens status could not be ascertained as it was not visible. Intraocular pressure measured digitally was low in both eyes. B scan ultrasonography of the posterior segment was unremarkable bilaterally.

**Figure 2:**
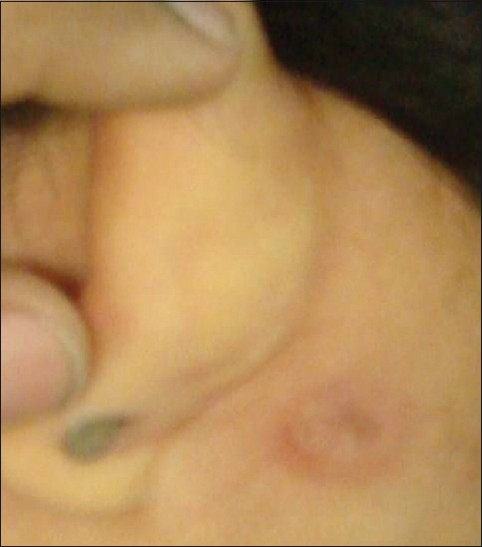
Ulcerated lesion with bluish undermined margins adherent to underlying post-auricular lymph node on the left side

Preauricular, post-auricular and cervical lymphadenopathy with scarring was noted. There was an ulcerated lesion with bluish undermined margins, adherent to the underlying post-auricular lymph node on the left side [Figure 2]. On systemic examination there was no clinically detectable abnormality in the chest, no rash or eczema, no purpuric lesion in any part of the body, and arthritic or joint deformities and hepatosplenomegaly were absent.

The skin lesions were evaluated by a dermatologist and were consistent with scrofuloderma. Laboratory tests included complete hemogram, screening for human immunodeficiency virus, renal and liver function tests, fecal examination for ova and cysts, Rheumatoid factor, antinuclear antibody, chest X-ray, Mantoux test (purified protein derivative), fine needle aspiration cytology (FNAC) of the lymph node with staining for acid fast bacilli, and histopathological examination of the aspirate. Corneal scraping was performed and samples obtained from the margins of the ulcer were sent for smear examination, microbial culture, and sensitivity screening.

The pertinent positive findings included an elevated Westergren erythrocyte sedimentation rate (ESR) of 58 mm and Mantoux test showed an induration of 40 mm at 48 h. FNAC of the lymph node showed evidence of necrosis along with the presence of lymphocytes and epitheloid cells consistent with tuberculosis, but the staining for acid fast bacilli was negative. The smear examination of the corneal scrapings revealed the presence of gram-positive cocci and microbial culture showed growth of coagulase-negative *Staphylococcus*. The culture for mycobacterium on Lowenstein Jensen medium did not show any growth.

The patient was treated initially with topical cefazolin sodium 5% and tobramycin sulfate 1.3% every hour; homatropine hydrobromide 2% eye drops four times daily. The patient was also treated with oral rifampicin, isoniazid, ethambutol, and pyrazinamide by the dermatologist. There was symptomatic improvement and a decrease in the area of infiltration after medical therapy. The corneal ulcer responded well; however there was subsequent formation of anterior staphyloma in both the eyes within 2 weeks [Figure 2]. She underwent tectonic keratoplasty in the right eye followed by the left eye 10 days later. The host cornea diameter was cut to 9 mm and the donor tissue was cut with a 10 mm corneal trephine keeping a recipient-donor disparity of 1 mm in both eyes. The specimen revealed stromal edema along with the presence of dense inflammatory exudates predominantly comprising of neutrophils, nuclear debris, and uveal tissue pigmentation on the endothelial layer of the cornea.

Eight weeks postoperatively her best corrected visual acuity improved to 20/200 in the both the eyes with clear grafts. There was no recurrence of infectious keratitis in any eye until the last follow up. The skin lesion also progressively reduced in size after starting anti-tubercular treatment.

## DISCUSSION

Scrofuloderma is a form of reactivation tuberculosis^6^ and is one of the most common forms of cutaneous tuberculosis. Usually it results from the tubercular process in the lymph nodes, bones, joints and/or the subcutaneous tissues, with formation of cold abscesses and a secondary breakdown of the overlying skin.^7^ It often occurs in children and patients with low immunity and involves most commonly the lymph nodes of the cervical region. There have been reports of ocular scrofuloderma with unilateral^4^ as well as bilateral^5^ orbital tuberculosis presenting with proptosis.

Tuberculosis with ocular involvement is likely to occur either by direct invasion or as a hypersensitivity reaction.^8^ Tuberculosis may involve any part of the eye and may appear in different clinical forms, which may be primary or secondary.^9^ In secondary ocular tuberculosis, infection results either from contiguous spread from adjacent structures or by the hematogenous route.^9^ Corneal lesions manifest as phlyctenulosis, infiltrates, ulceration, and interstitial keratitis. An association between phlyctenulosis and tubercular protein hypersensitivity has been established by both epidemiological and experimental data.^10^

The history of recurrent quadrantic redness in both eyes was relieved by topical steroids suggesting that our patient had recurrent bilateral phlyctenulosis. Lymph node histology was suggestive of primary focus of tuberculosis and the presence of positive Mantoux test indicated towards high tubercular protein hypersensitivity.

Patients with phlyctenulosis are treated with topical steroid. This may reduce local immunity and may predispose the eye to infection. The micro-organism detected in the corneal scraping in our case was coagulase-negative *Staphylococcus* which may be present as a normal commensal in the eye and may cause ulceration in eyes with reduced local immunity. In the present case, there is a strong possibility of the occurrence of multiple areas of phlyctenulosis which were infected and resulted in the formation of multiple corneal ulcers, which did not respond to medical therapy and subsequently perforated at all sites. The central perforation noted in this case is the result of contiguous paracentral and central spread of an uncontrolled infection. As the histopathology failed to reveal evidence of mycobacterium and the corneal scraping performed previously did not reveal mycobacterium, a diagnosis of primary tubercular keratitis was highly unlikely.

Penetrating keratoplasty was performed in both the eyes for tectonic purposes as well as to make the patient ambulatory. Although the graft was clear and the patient had reasonably good vision considering the indication for which the surgery was performed, the risk of a similar episode in future cannot be ruled out. Hence this patient requires strict and regular follow up due to the risk of future episodes and the risk of graft rejection due to the large diameter graft.

The key to diagnosis of ocular tuberculosis is a high index of suspicion especially in tropical countries due to its non-specific and protean manifestations. Scrofuloderma may have various ocular associations. There is definite role of steroid in cases of phlyctenulosis; however, judicious and supervised use of topical steroid is required to decrease the risk of occurrence of corneal ulcer which may be refractory to medical management. This risk is increased in a female during pregnancy and in the early postpartum phase when immunity is somewhat compromised.
